# Underlying socio-political processes behind the 2016 US election

**DOI:** 10.1371/journal.pone.0214854

**Published:** 2019-04-09

**Authors:** John Bryden, Eric Silverman

**Affiliations:** 1 School of Biological Sciences, Royal Holloway, University of London, London, United Kingdom; 2 The London College of Political Technologists, Newspeak House, London, United Kingdom; 3 MRC/CSO Social and Public Health Sciences Unit, University of Glasgow, Glasgow, United Kingdom; Universidade Estadual de Maringa, BRAZIL

## Abstract

Recently we have witnessed a number of rapid shifts toward populism in the rhetoric and policies of major political parties, as exemplified in the 2016 Brexit Referendum, 2016 US Election, and 2017 UK General Election. Our perspective here is to focus on understanding the underlying societal processes behind these recent political shifts. We use novel methods to study social dynamics behind the 2016 Presidential election. This is done by using network science methods to identify key groups associated with the US right-wing during the election. We investigate how the groups grew on Twitter, and how their associated accounts changed their following behaviour over time. We find a new external faction of Trump supporters took a strong influence over the traditional Republican Party (GOP) base during the election campaign. The new group dominated the GOP group in terms of new members and endorsement via Twitter follows. Growth of new accounts for the GOP party all but collapsed during the campaign. While the Alt-right group was growing exponentially, it has remained relatively isolated. Counter to the mainstream view, we detected an unexpectedly low number of automated ‘bot’ accounts and accounts associated with foreign intervention in the Trump-supporting group. Our work demonstrates a powerful method for tracking the evolution of societal groups and reveals complex social processes behind political changes.

## Introduction

Donald Trump’s victory in the GOP primaries and the Presidential race surprised political analysts and confounded pollsters. Trump achieved this victory via a populist campaign which incorporated racially-charged and misogynistic language [1, 2]. This unusual campaign shifted the direction of the GOP and the US right-wing toward the far-right of the political spectrum [2]. An important factor behind this success was the campaign’s use of social media communication channels, especially Twitter [3].

Twitter, and social media in general, have become important tools for politicians and their followers to spread political messages [4–8]. The hierarchical structures commonly found in social media networks mean that well-connected politicians act as hub nodes, with information and influence spreading outward over the network. Political parties form clusters which reside at the centre of these networks [5, 8–10]. As intra-party discourse is increasingly taking place online, traditional boundaries between politicians, activists, party-members and members of the public have become increasingly blurred.

The increased openness of parties moving their political discourse online has undoubted benefits for transparency and accountability. The concern is the opportunity this provides for an external group to target the online presence of a political party, and then start to dictate their political direction. The ability of a minority group to rapidly generate a new political faction and take control of a major political party in this way can cause problems for democracy [11, 12]. Such a novel process would differ radically from more traditional models of dynamics amongst political elites who occupy different internal factions of a party [11, 13], moving to a model whereby external factions play a stronger role [13–15]. In this work we look for evidence of an external group influencing the online presence of the GOP in the run up to the 2016 election and how this can explain the shift in the party’s direction.

Recent changes in the rhetoric and policy platform of the GOP provide an example of the impact of external groups. During the 2016 election cycle, the Alt-right—an online community of activists who identify as white nationalists—emerged as a major influence on GOP political discourse. The Trump campaign and pro-Trump media outlets like Breitbart News co-opted Alt-right rhetoric, provoking a movement from implicit racial priming in campaigns to explicit racial messaging [2]. Trump and his supporters were able to use the shock value of explicit racial rhetoric to gain media attention for his platform, without destroying the GOP’s electoral calculus. The Trump campaign marked a large and sudden shift in rhetoric and target demographics in 2016, unprecedented in modern US political history [16].

Our work seeks to understand how communities of activists might provoke such a significant shift in the attitudes and rhetoric of a major political party. We looked for how political activists organised themselves and their political messaging, and how these aspects changed over time. We did this by examining Twitter data in the context of the 2016 election, given that Twitter has been shown to reflect US national polling aggregates accurately [17]. By studying these changes, we can analyse and document shifting allegiances during the election cycle, and the roles of different factors driving these dynamics.

## Results

**Fig 1:**
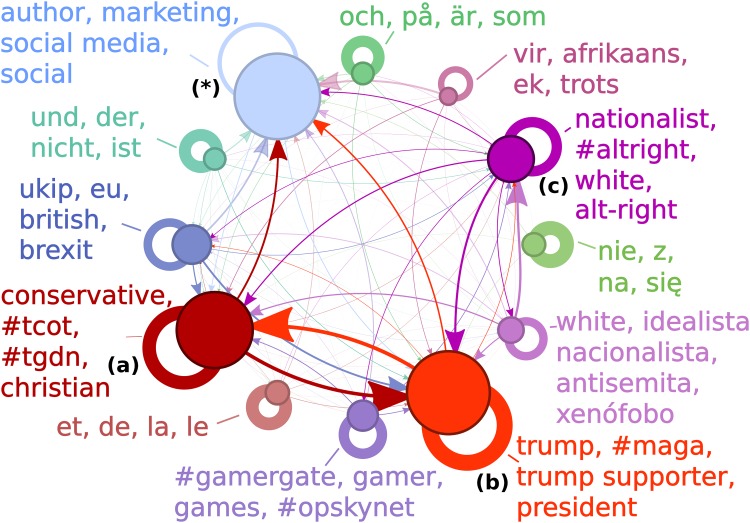
Map of far right groups sampled. We found three distinct groups of accounts associated with Trump’s campaign. These are emphasised with stronger colours:**(a)** GOP group, **(b)** Trump group, and **(c)** Alt-right group. The plot shows all groups (> 2000 members) found by our sample linked according to how often their members follow accounts in other groups, and annotated with words used significantly commonly in the biographies of the group’s members. The thickness of links was calculated by recording, for each member of the originating-group, the proportion of accounts which were followed in the linked-group, and then taking an average of these proportions. Links are the same colour as the group containing the following accounts and point to the group containing the followed accounts. The most cohesive group (thickest self-loop) is the Trump group with an average member having 70% of the accounts they have followed being in the same group. The largest group was size 55,730, (marked *).

Our data gathering approach was designed to focus on accounts related to right-wing US politics. To do this we designed our sampling technique around the principle that accounts of similar political allegiance tend to follow each other [7, 18] and sampled approximately 250,000 Twitter accounts. We then identified groups within our sample where members of the same group tended to follow one another [19], a method shown to find groups with shared political interests [7]. To find those interests we automatically characterised each group using biographical information on member profiles [18] (see Methods). This approach identified a number of right-wing groups based in and outside of the US. We generated a picture of how members of the groups follow one another (see Fig 1 for groups > 2000 members and S1 Fig for groups > 200 members).

**Fig 2:**
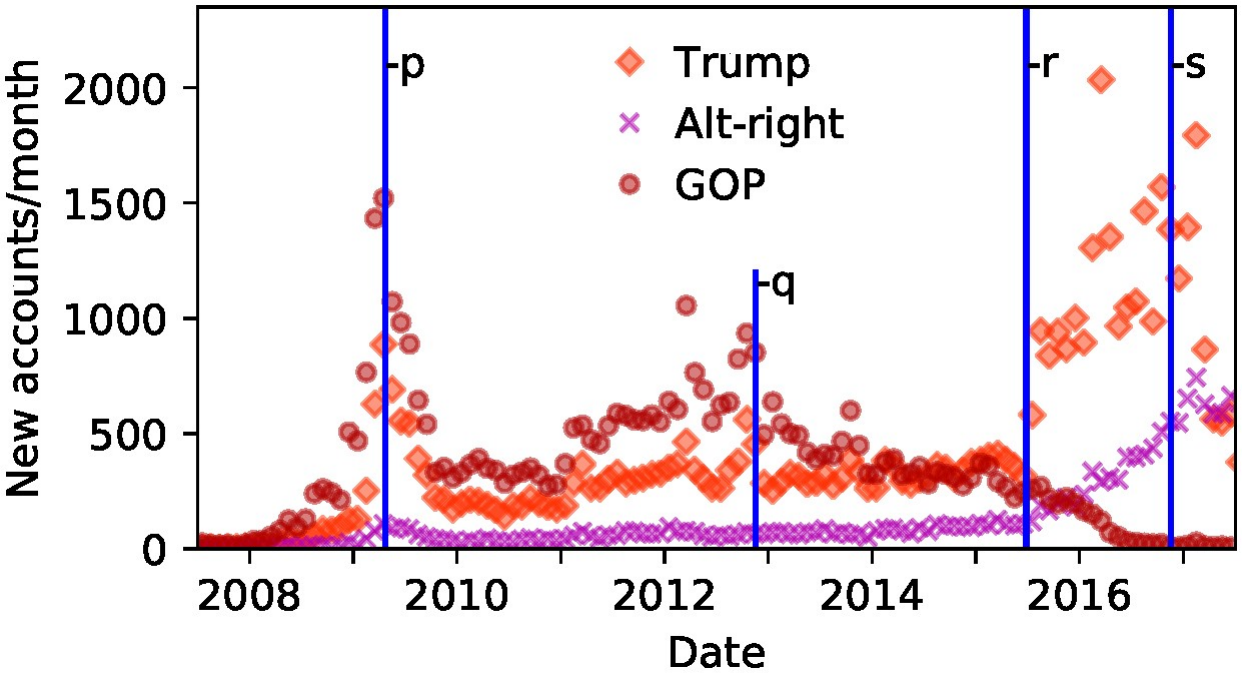
Changing growth rates of each group. Shows how a decrease in new accounts aligned with the GOP group coincided with a growth in the Trump and Alt-right groups, especially after Trump’s campaign announcement in June 2015. We plot the growth rates over time of the three focal groups: Trump (orange diamonds), Alt-right (purple Xs), and GOP (red circles). Events shown: **p**, Tax Day Events on 12 April 2009 associated with the Tea Party Movement; **q**, 2012 US elections on 6 November 2012; **r** Trump’s election campaign announcement on 16 June 2015; **s** 2016 US elections on 5 November 2016.

We focused in greater detail on the three focal groups which we identified in Fig 1. We would expect that the members of these groups joined Twitter due to certain political events, plotting the rate of new accounts created against time for each group demonstrates this in more detail (see Fig 2). The figure shows how all three groups were influenced by particular events including the Tea Party movements of 2009, the presidential election of 2012 and Trump’s campaign launch in mid-June 2015. After the 2012 presidential election growth of the GOP group stagnated. After Trump’s campaign announcement growth of the GOP group stalled while growth of the Trump group accelerated.

**Fig 3:**
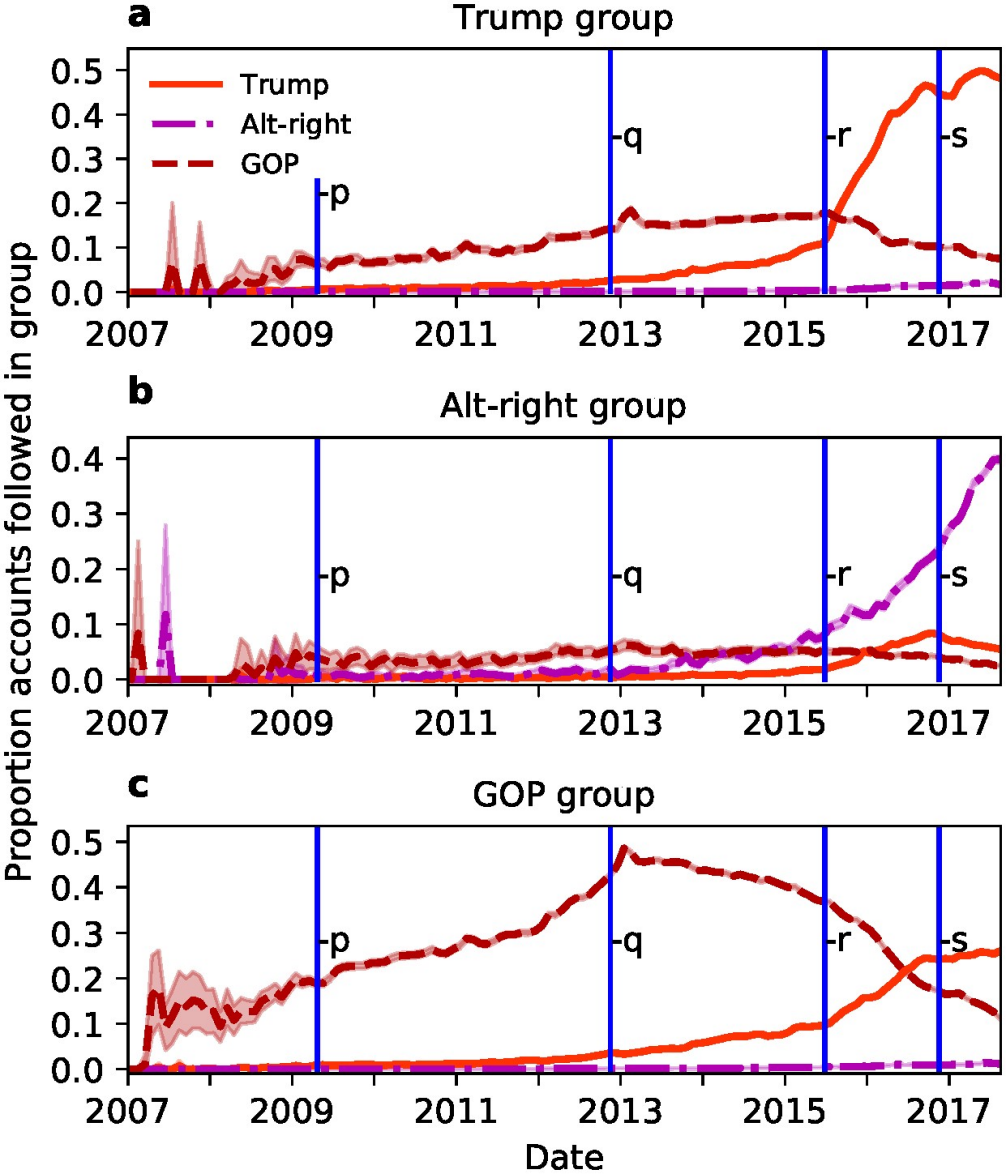
Accounts followed shifted from GOP to the Trump group. After the 2012 election, there was a shift from following accounts in the GOP group to following accounts in the Trump group (panels **a**, **b** and **c**). Members of the Alt-right group have increasingly followed one another over time (panel **b**), but are not followed by GOP or Trump group members (panels **a** and **b**). Each of the three panels show how the members of one focal group followed members of the groups over time. The time traces show the proportions of accounts that were followed in the Trump (orange line), Alt-right (purple dash-dotted line), or GOP (red dashed line) groups, averaged over each member in the originating group. Events shown (**p**, **q**, **r**, and **s**) are as in Fig 2. The data points have very small 95% confidence intervals (shaded areas, see Methods) which demonstrates that this pattern is highly statistically significant: The members of a group largely changed their following-behaviour in concert.

To better understand the decrease in growth rate of the GOP, we looked for evidence of an ideological shift. Political ideology can be inferred by examining which political actors a Twitter user is following [20]. Consequently, we looked at how the following behaviour of the group members changed over time. For each member of one of our groups, we estimated the proportions of accounts in each group which were followed by that member, on a monthly basis (see Methods for how we inferred following dates). The per-member average proportion of accounts followed between the groups is shown in Fig 3. We animate these patterns of following behaviour, along with the group sizes over time, in a video (see S1 File). The figure shows how, over time, there is a shift from members of the Trump and GOP groups both following members of the GOP group to both following members of the Trump group. This indicates a shift in ideological position of the party to that associated with the Trump group.

A few concerns may be raised about the quality and breadth of our data, which we now address. It has been widely proposed that computational propaganda from automated Twitter accounts (bots) and/or foreign intervention may have played a strong role in developing Trump’s online following [21, 22]. A consequence of this may be that our data may be mostly comprised of such bots. Furthermore, the accounts we have found are relatively few in comparison to the US electorate. To address these points we estimated the numbers of bots in each group, and calculated the number of followers on the whole of Twitter which follow at least one account in each group (see Table 1, and S2 Fig for distributions). The numbers of bots is low, and there is a greater proportion of bots in the GOP group than the Trump group. This is not consistent with the idea that the Trump group was generated using automated accounts. Further, our tests of the entire sample indicate that less than 200 out of some 250,000 sampled accounts have been flagged as being accounts from Russia [23]. Consequently, we argue our data contain a large number of genuine Twitter accounts formed into cohesive groups. Due to their large numbers of followers, these accounts will have acted as amplifiers for the messaging of Donald Trump’s campaign and are likely to have had a profound influence on the primary and presidential elections in 2016.

**Table 1:** The three focal groups have few bots and are widely followed by other accounts on Twitter. We estimated numbers of bots by testing 2,000 accounts using Botometer [24] (scores >0.8 were deemed bots). The number of followers was the number of unique accounts on the whole of Twitter that had followed at least one account in each group. The number of followers for all three groups combined is 57 million, comparable in size to the 63M who voted for Trump in the election. Figures are given to 2 d.p., K means thousands, and M means millions.

Group	Size	Bots	Followers
GOP	44K	4.0%	38M
Trump	52K	1.5%	25M
Alt-right	16K	0.71%	9.2M

## Discussion

The method presented here shows how it is possible to uncover underlying socio-political processes behind real political change. This is done by first identifying online groups, and then observing how they grow over time and how their members change the other accounts they follow. Political changes reflect these underlying processes so the ability to observe them is critical. Consequently, this approach is likely to be of great value in the social and political sciences. Twitter’s important role in party political communication and activism means that we can use this method to study shifting political discourses in a way we never could before, enabling us to develop more powerful theories of socio-political dynamics.

To study social and socio-political processes, our method incorporates two key novel aspects. First, an innovative sampling procedure which allows us to target and download key groups of interest. Second, our generated data shows how groups evolve over time, including the shifting allegiances of group members. Since these data are dynamic networks, this approach can enhance our understanding of dynamic social processes. While much modelling work has been done [25–29], the next step is to bring these models to data such as that presented in this work [30–33].

Given the self-selecting nature of Twitter groups, we may be concerned that the groups we have found may not truly reflect the position of the party’s base. However, the high levels of intra-group connections are representative of an interconnected political party [7, 18]. The behaviour observed in this sample during the 2016 election cycle does match the behaviour seen during the rise of the Tea Party and victory of Trump in the primary elections. The sample here was targeted initially at the Alt-right, but we found connected accounts which were also associated with the right-wing of both the US and other countries, suggesting that the sample has captured a wide swathe of right-wing political communication and not just fringe groups or extremist individuals. The sample also went beyond the right-wing political sphere and sampled a loosely intra-connected group of general Twitter accounts (see group marked * in Fig 1), indicating that it had captured a comprehensive picture of those accounts associated with the US right-wing.

Social media has continued to expand its influence over the political process [4–10, 17]. These technologies enable individuals to easily connect with one another, based on shared political opinions. It follows that they are likely to be playing a strong role in recent social-political movements by allowing politicians to rally disaffected individuals. At the very least, social media data allow us to observe the processes behind changing political factions. Understanding these processes, and how they happen is critically important to understanding modern democracy and voter behaviour, and our method marks a step change in how political factions can be identified, analysed, and tracked.

The data we have presented show how, starting from June 2015, an external faction shifted the GOP away from its previous base. This shift could be explained by results from another study, which indicate that as support for an extreme position increases, support for a corresponding less extreme position will increase with a nonlinear relation [28]. Reasons for the emergence of the Trump group are less clear because it is relatively isolated from the more extreme alt-right group. There is, however, a potential role played by automated accounts where even a small number of bots were shown to have been able to shift opinion in Trump supporters [34]. That said, our data suggest that Trump’s followers consist largely of real, highly-engaged supporters.

An alternative perspective looks at psychological reasons behind the shift to the right. Evidence shows that poor levels of well-being and low optimism for the future played a strong role in the shift toward Trump [35], with economic insecurity and a cultural backlash being key factors [2, 16, 36]. In this case, our results would indicate that disaffection for a major political party may have provided space in which a new political movement could grow and eventually take over that party.

## Conclusion

Donald Trump’s ascent to the Presidency has prompted a great deal of effort amongst pollsters, political scientists and social scientists to unearth the reasons for his unexpected success. Here we provide a method to follow the shifts in group membership and influence that can occur in political parties, and in so doing provide indicators of impending moves toward extremism within those parties. Our results fit into a picture where the Trump campaign’s mobilisation of a targeted group of supporters more than made up for Clinton’s funding advantage [29, 37]: A significant shift in the US political landscape. With that in mind, developing a more robust understanding of how political factions can be identified and analysed can give us a way to follow these fast-appearing and highly-motivated supporter groups, and their influence on politics.

## Methods

### Ethics

We were given ethical approval for the data collection and storage methods used in this research by the relevant Departmental Ethics Committee at Royal Holloway University of London. Even though all the information downloaded from Twitter is public, it was stipulated that we would only publish anonymous and/or agglomerate data. Consequently, we are only releasing the Twitter IDs which we sampled for the study and the agglomerate data published in the manuscript.

### Downloading a sample of accounts

Our aim was to bias our sample to download accounts which had a right-wing political orientation, as defined in the American political context. To do this, we developed a novel weighted snowball-sampling technique in order to download a sample of twitter accounts which all share similar interests. Given our intent to focus on right-wing groups in the US, we began the sample with a prominent account followed by a large number of Alt-right and Trump supporters: the ‘_altright_’ Twitter account. Our technique biases the sample to accounts that are more likely to follow accounts which have already been sampled, collecting groups of close-knit accounts which are closely linked to those accounts already sampled. The process incrementally builds a dataset of accounts starting in a local community and then on to other closely linked communities. Other community-sampling techniques focus on a single group [38].

We downloaded sample accounts using the Twitter REST API. For each account sampled we recorded its creation date, biography, and the lists of which accounts they had followed and which accounts had followed them. People on Twitter tend to follow other accounts quite broadly but when two accounts follow one another that is a much better indication that they have something in common. Other approaches have used retweet or mention networks [7, 18], however we decided to use a follower network because these links are more permanent and less transient. For these reasons we used mutual following to build our network of accounts.

To download our sample, we maintained a master list of accounts to be sampled, which each account being assigned a score determining which would be the next to be sampled. For any new account sampled, we generated a set of all accounts which both followed and were followed by this new account. This set of mutual-follower accounts was merged with the master list of accounts and their sample scores were updated. Score updates were done by dividing the score of the account which had been sampled equally amongst its mutual followers. The initial account was assigned a sample score of 1.0 and all other accounts were initiated with a sample score of 1.0. After the scores were updated, we then identified the highest-scoring account on our list which had not yet been sampled, and iterated the procedure with that account.

### Generating and characterising groups of accounts

Our sample generated a network of accounts with accounts linked to one another based on whether they had mutually followed one another. We generated community structure for these accounts using the Louvain Method [19] and used groups at the lowest level of the hierarchy. The groups found were characterised by generating word frequencies for every word and pairs of words (unigrams and bigrams) used in the biographies of the Twitter accounts for each group. Words were converted to lower case and stripped of punctuation.

In order to establish which words or word pairs characterise each group, we compared the fraction of users that use each word within a group with the fraction of users that used the word globally [18]. We then assessed how unlikely it was that the difference between these two fractions could have happened by random chance. This is given by the standardised *Z*-score which, for each word/word pair used in community *c*, is

$$\begin{array}{c} Z=\frac{\mu_{c}-\mu_{g}}{\sigma_{g}/\sqrt{N_{c}}} \end{array}$$

where *μ*_c_ is the fraction of users in community c which have used the word, *μ*_g_ is the fraction of all users that have used it, *N*_c_ is the number of users in community c, and *σ*_g_ is the standard deviation of usage of the word amongst all users

$$\begin{array}{ccc} \sigma_{g}^{2} & = & \frac{1}{N}(\mu_{g}N{\left(1-\mu_{g}\right)}^{2}+\left(1-\mu_{g}\right)N\mu_{g}^{2})\\ & = & \mu_{g}\left(1-\mu_{g}\right) \end{array}$$

where *N* is the global number of users. The words with the highest *Z*-Scores are those which are used more often by and are used to characterise that group.

We ran an initial sample in November 2016, shortly after the Presidential election. To confirm the results we found had not been by chance, we reran the sample in July 2017 starting with the same initial ‘_altright_’ account. However, Twitter had suspended the account in the interim, so we used a downloaded copy of the account from November 2016 including its list of followers and used this to seed the sample. The rerun of the process yielded similar results to the first run.

The sampler found a group with a large number of relatively neutral accounts which were also loosely interconnected (see starred group in Fig 1). Due to the nature of our sampler, one would expect to find that, after it had sampled the majority of interconnected accounts associated with the wider right-wing group, it would then start to sample accounts from the remainder of the Twitter web site. These accounts would not be closely interconnected but would be relatively isolated from the other right wing groups. The biographies of the group members were also consistent with this picture of the accounts not having strong political affiliations. Due to the fact that our sampler had sampled a large number of accounts from this looser-knit group and more-neutral group, we were confident that our sample had covered a broad sample of right-wing affiliated groups.

### Inferring account-following dates

In order to track how accounts shifted their following behaviour over time, we needed to develop a method to infer the date of each following-event (i.e., when an account followed another account). Though our data contained a list of accounts followed, the order in which they were followed, and account creation dates, we did not have the specific dates of following-events. However, note that it is not possible to follow an account until it has been created. We used this information to infer that the following-event of an account must have happened after it was created, and consequently that all subsequent following events in the list must have happened after that date as well. In the list of accounts-followed we marked *timestamp-accounts* by going through the list in the order which they were followed (starting at the earliest account) and marking those accounts which had been created after all the previous accounts in the list. All accounts followed in the list after these timestamp-accounts must have been followed at some time after the timestamp-account was created. We inferred that the timestamp-accounts had been followed on their creation date. We then inferred the date-followed for accounts listed between the timestamp-accounts by spacing these dates evenly between these creation dates.

### Statistical analysis

Our data show how the members of different groups change the way they follow accounts in the other groups over time. We wanted to statistically confirm that this following-pattern had not happened due to some random process in the following behaviour of a group’s members. Consequently we looked at the accounts followed by members of a group on a monthly basis. For the accounts followed by each member each month, we then calculated the proportions of these followed-accounts according to the group membership of the followed-accounts. We then calculated the mean and corresponding 95% confidence intervals for these per-group proportions. Because the 95% confidence intervals were relatively very small, we concluded that the patterns we found were highly statistically significant and did not need further testing to support our argument.

## Supporting information

S1 Fig All groups in the sample. Plot showing the groups found by our sample and summarising the how the accounts follow one antoher at a group level. Groups shown (> 200 members) are sized by the number of members. Lines between groups are the same colour as the originating group. Links are the same colour as the group containing the following accounts and point to the group containing the followed accounts. The thickness of the line represents the average proportion of accounts, per individual in the originating group, which are followed in the linked group.
(PNG)

S2 Fig Botometer distributions. The distributions of scores assigned by Botometer to samples of 2,000 accounts taken from each of the three focal groups.
(PNG)

S1 File Group dynamics. Movie showing the dynamics of the three groups and their following behaviour over time. It shows how initial growth and internal following behaviour of the GOP group is superceded by growth and following of the Trump group. Areas of groups represent the size of the group at the time shown. For each month, link widths are scaled to represent the average proportion of accounts, per individual in the originating group, which were followed in the linked group. Links are the same colour as the group containing the following accounts and point to the group containing the followed accounts.
(MP4)
