# Supplementary figures and images for: Underlying socio-political processes behind the 2016 US election
Source: PLoS One. 2019 Apr 9;14(4):e0214854. doi: 10.1371/journal.pone.0214854 (PMC6456177; doi:10.1371/journal.pone.0214854)

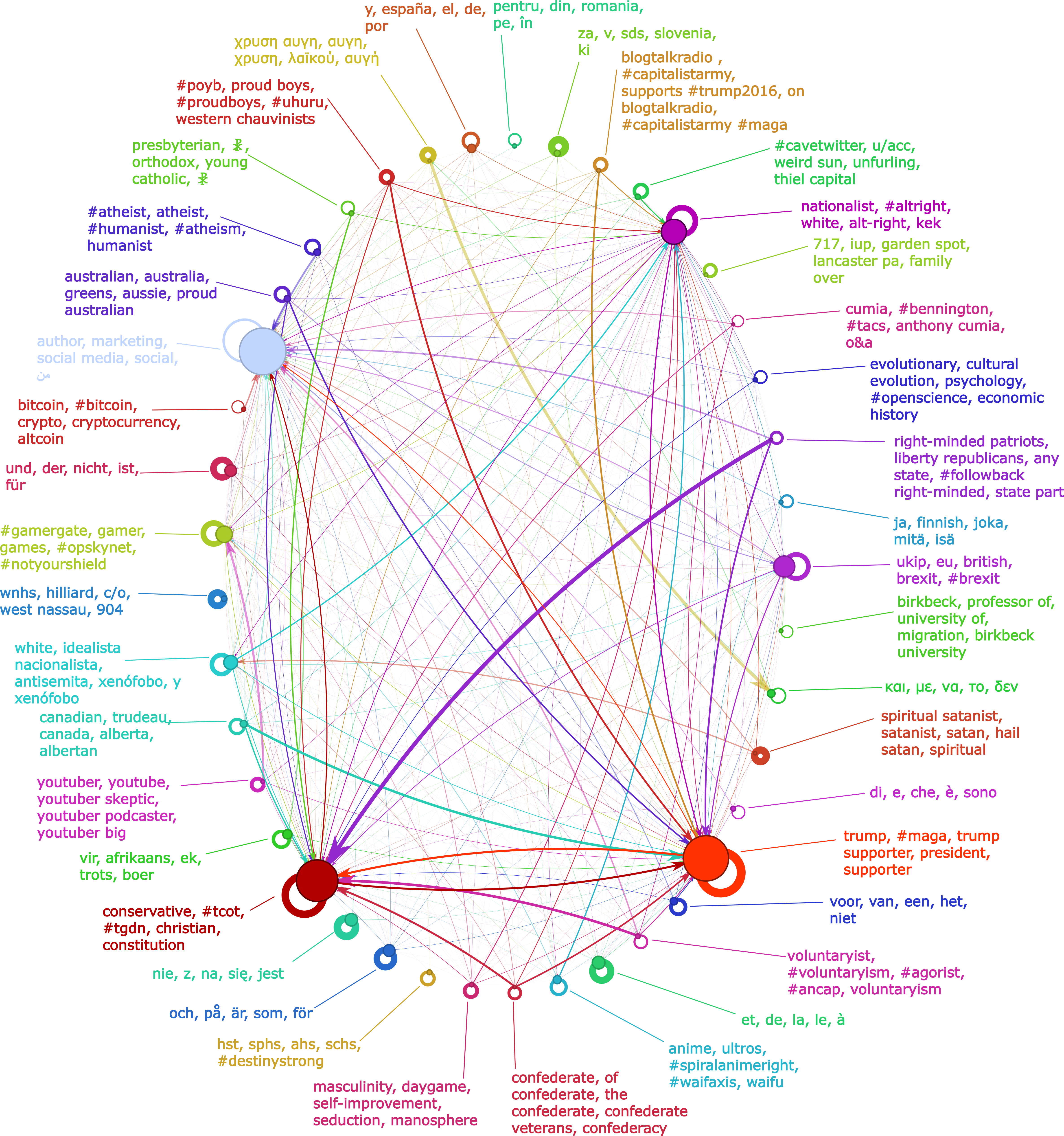

Supplement: S1 Fig — Plot showing the groups found by our sample and summarising the how the accounts follow one antoher at a group level. Groups shown (> 200 members) are sized by the number of members. Lines between groups are the same colour as the originating group. Links are the same colour as the group containing the following accounts and point to the group containing the followed accounts. The thickness of the line represents the average proportion of accounts, per individual in the originating group, which are followed in the linked group. (PNG) [file pone.0214854.s001.png]

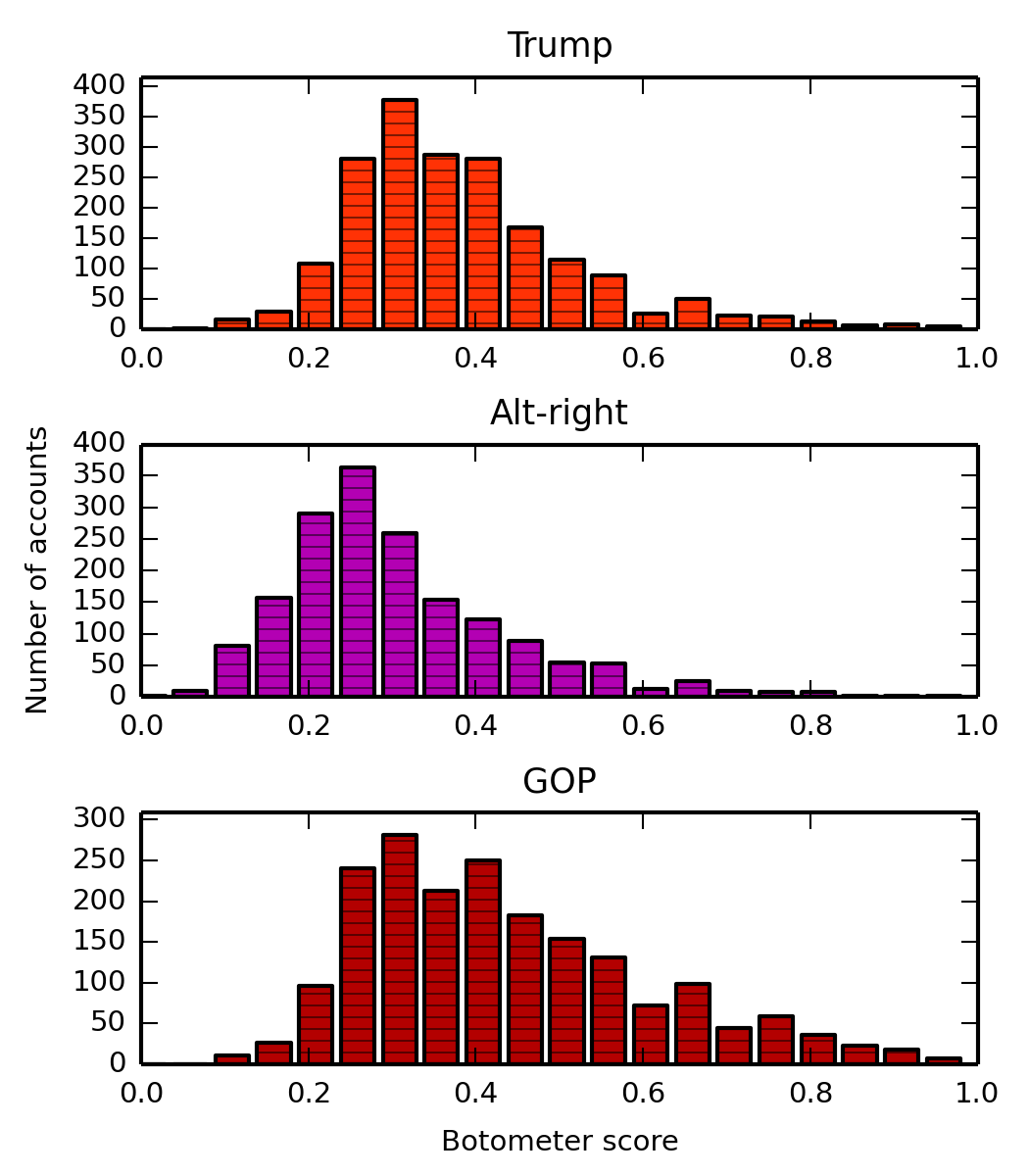

Supplement: S2 Fig — The distributions of scores assigned by Botometer to samples of 2,000 accounts taken from each of the three focal groups. (PNG) [file pone.0214854.s002.png]
